# Clinical outcomes of continuous local antibiotic perfusion in combination with debridement antibiotics and implant retention for periprosthetic hip joint infection

**DOI:** 10.1038/s41598-025-11808-y

**Published:** 2025-07-18

**Authors:** Yuta Hieda, Hyonmin Choe, Akihiro Maruo, Koki Abe, Masashi Shimoda, Hiroyuki Ike, Ken Kumagai, Naomi Kobayashi, Yutaka Inaba

**Affiliations:** 1 Department of Orthopaedic Surgery, Yokohama City University, 236-0004 3- 9 Fukuura, Kanazawa-ku, Yokohama City, 236-0004 Kanagawa Japan; 2 Department of Orthopaedic Surgery, Harima Himeji General Medical Center, 3-264 Kamiya-cho, Himeji City, 670-8560 Hyougo Japan; 3 Department of Orthopaedic Surgery, Yokohama City University Medical Center, 4-57 Urahune-cho, Minami-ku, Yokohama City, 232-0024 Kanagawa Japan

**Keywords:** Continuous local antibiotic perfusion, Debridement, Implant retention, Periprosthetic joint infection, Orthopaedics, Bacterial infection

## Abstract

Periprosthetic joint infection (PJI) is a complication of peri-implant biofilm-based treatments and confers resistance to antimicrobial therapy. Integrating continuous local antibiotic perfusion (CLAP) with conventional surgery for PJI facilitates the local delivery of low-flow, high-concentration antimicrobials. This study aimed to evaluate the efficacy and safety of CLAP for treating PJI. This study included patients diagnosed with hip PJI who underwent debridement, antibiotics, and implant retention (DAIR) augmented by CLAP. Gentamicin was administered at a high concentration (1.2 mg/mL) and low flow rate (2.0 mL/h for 24 h). We evaluated implant survival and complication rates associated with adding CLAP to conventional DAIR surgery. Of the 22 patients, including 11 with chronic infection, DAIR surgery supplemented with CLAP resulted in implant survival in 20 patients (90.9%). In contrast, among 10 patients treated with DAIR without CLAP (non-CLAP group), implant survival was 70%. The mean follow-up period was 42.6 ± 31.5 (range, 12–161) months in the CLAP group and 56.8 ± 28.8 (range, 28–114) months in the non-CLAP group. During CLAP treatment, renal function worsened in two patients; however, it improved rapidly after CLAP completion and device removal. No major complications were observed. CLAP demonstrated promising results in treating acute and chronic PJI. However, monitoring and regulating blood antimicrobial levels is crucial to avoiding renal dysfunction. CLAP is a treatment option for PJI that can destroy bacterial biofilms.

## Introduction

### Background

Periprosthetic joint infection (PJI) is a complication of arthroplasty. It is characterized by bacterial biofilms on implants, which render systemic antimicrobial therapy ineffective^1–4^. The minimum biofilm eradication concentration (MBEC) required is often 100 times higher than that required to inhibit bacterial growth alone^5,6^. Surgical interventions, such as two-stage revision surgery for removing infected implants, frequently result in considerable muscle weakness and reduced mobility^7^. PJI treatment includes intravenous and local antibacterial treatments, with the latter being crucial for implant preservation by delivering antimicrobials at concentrations above the MBEC^8^.

Antimicrobial-containing polymethylmethacrylate is utilized for targeted, high-concentration antibiotic delivery^9^. However, maintaining a consistent dose with local antibacterial treatments is challenging, and postoperative dose adjustments are not feasible^10^. Continuous local antimicrobial perfusion (CLAP), used alongside conventional surgery, provides an effective infection management strategy by ensuring high-concentration, low-flow antimicrobial delivery^11–15^. CLAP implementation, facilitated by specific devices, allows for postsurgical adjustment of concentration and flow rate, maintaining consistent local antimicrobial dosing. However, the antibiotic release from polymethylmethacrylate may decrease over time^10^.

### Rationale

CLAP may enhance biofilm destruction and implant preservation in chronic PJI. However, no studies have evaluated its efficacy and safety in PJI. This study aimed to evaluate implant survival and complication rates in patients with PJI who underwent debridement, antibiotics, and implant retention (DAIR) surgery supplemented with CLAP.

### Patients and Methods

#### Study design and setting

We conducted a retrospective chart review to identify and enroll patients diagnosed with PJI of the hip who underwent DAIR with CLAP between December 2010 and July 2023 at our institutions and without CLAP between March 2013 and February 2021 as the historical control group, and the participants were classified into the CLAP and non-CLAP groups. The institutional review board of Yokohama City University (No: 230900002) approved this retrospective study. All the procedures were performed in accordance with relevant guidelines. Written informed consent regarding potential complications was obtained at the time of CLAP treatment, and an online opt-out form was used for data collection. Approval for the off-label use of the CLAP tubes was obtained from the Ethics Committee. All procedures were conducted in accordance with the Declaration of Helsinki.

#### Participants

DAIR with CLAP was performed in all patients with PJI without implant loosening, provided the CLAP device was ready on the day of surgery. Patients with PJI whose implants had septic loosening were indicated for one or two-stage revision surgery. Patients with chronic PJI infection without the implants loosening also underwent DAIR upon request rather than a two-stage revision if the patient’s condition, range of infection, and causative organisms were identified. This study included only patients who had undergone surgery at least 1 year postoperatively. Patients who died over 1 year postoperatively due to causes unrelated to PJI or CLAP complications were included, whereas those who died within 1 year postoperatively due to factors unrelated to surgery were excluded.

We utilized the diagnostic criteria from the 2018 International Consensus Meeting on PJI, which defines PJI based on either major criteria or a score of ≥ 6 points^16^. For this purpose, we investigated the following: the presence of a fistula, number of specimens positive for bacterial culture, phase of infection (acute or chronic), preoperative serum C-reactive protein level, D-dimer level, erythrocyte sedimentation rate, neutrophilic infiltration on postoperative pathology, and intraoperative abscess formation.

#### Description of treatment or surgery

During DAIR surgery, bone and soft tissue were debrided using 0.35% saline-diluted povidone-isodine. Easily replaceable implants, such as sliding surfaces, were replaced as feasible. CLAP was performed according to the following procedure in the CLAP group, and drains were inserted only in cases considered necessary in the non-CLAP group.

##### Intraoperative administration route for CLAP

**Fig. 1:**
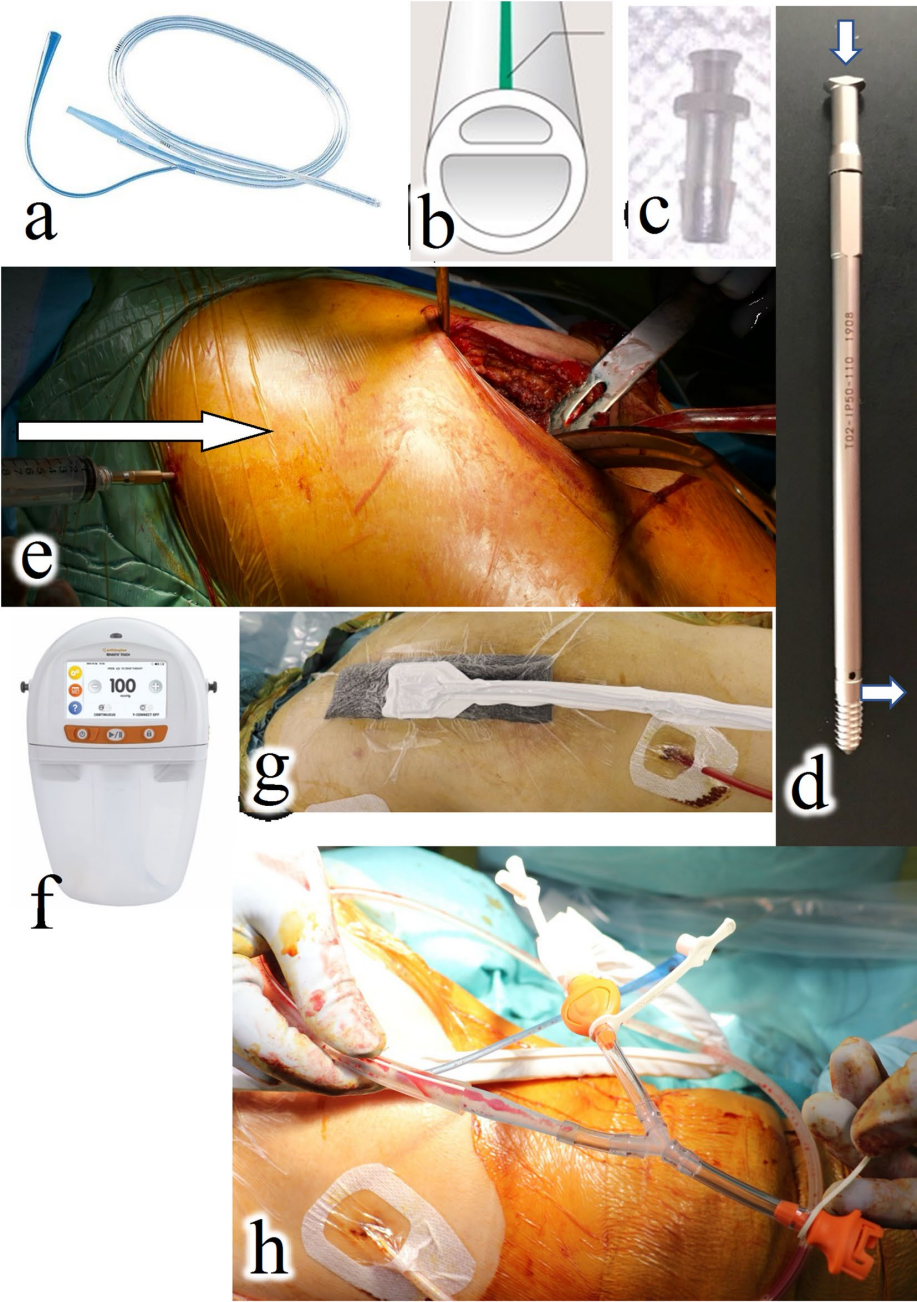
Device components of the continuous local antibiotic perfusion treatment system are shown. (**a**,** b).** Salem Sump tube (Cardinal Health K.K., Tokyo, Japan). This double-lumen tube allows for antimicrobial administration on one side and negative pressure application on the other. It features multiple holes for suction under continuous negative pressure to prevent blockage and is used for intra-soft tissue or intra-joint antibiotic perfusion. (**c).** Connector VRF606 (ISIS Co., Ltd., Osaka, Japan). The nasogastric tube is connected to the Salem Sump tube, constructing a route for antimicrobial administration. (**d).** An intramedullary antibiotic perfusion pin (Cubex Medical, Tokyo, Japan). These pins are fixed to the cortical bone, enabling antimicrobial administration into the bone marrow, as indicated by the white arrow. (**e).** Intramedullary antibiotic perfusion pin insertion. After insertion into the ala of the ilium, saline is injected intraoperatively (white arrow), while saline dispersal around the acetabulum is verified. (**f).** RENASYS TOUCH (Smith & Nephew, Watford, United Kingdom) system. It applies a continuous negative pressure of 60–80 mmHg to perfuse and collect local antimicrobials. The drainage fluid will be discarded. (**g).** Application of foam filler (Smith & Nephew, Watford, United Kingdom). Placed immediately above the wound, it applies continuous negative pressure to perfuse the wound, administering antimicrobials through the right tube while simultaneously collecting drainage fluid. **(h).** Y-connector branch tip (Smith and Nephew, Watford, United Kingdom). The tip is cut and connected to a Salem Sump tube for continuous negative pressure suction.

Intra-soft tissue antibiotic perfusion (iSAP): A Salem Sump tube (Cardinal Health K. K., Tokyo, Japan) > 20 Fr (Fig. 1a–c) was used. This tube has a double-lumen structure, allowing antimicrobial administration from one side and exudate collection under negative pressure from the other. After debridement, Salem Sump tubes were placed intraoperatively within the infected soft tissue, such as in the upper or lower layers of the fascia. This tube was originally designed for use as a gastric tube. Its application in this treatment constitutes an off-label use. This method was employed only after obtaining comprehensive informed consent from all patients.

Intra-joint antibiotic perfusion (iJAP): Salem Sump tubes were placed in the hip joint to perfuse the joint with antibacterial agents and eradicate the biofilm around the implants, guided by preoperative computed tomography imaging or intraoperative findings.

Intramedullary antibiotic perfusion (iMAP): An iMAP pin (Cubex Medical, Tokyo, Japan) was used. This hollow pin allows the injection of antimicrobials from one end and discharge on the tap side for cortical bone fixation and antimicrobial administration into the bone marrow (Fig. 1d). In cases of osteomyelitis identified preoperatively, an iMAP pin was inserted into the infected bone marrow to facilitate antibacterial perfusion (Fig. 1e). Bone holes were created using a Kirschner’s wire to establish an effective perfusion system to enhance the delivery of antimicrobials from the infection site in the suction direction.

##### Wound dressing

The wound was sparsely sutured during surgery to ensure complete dermal penetration of the antimicrobials administered using the CLAP device and to apply negative pressure to the wound effectively. Immediately after wound closure, the RENASYS TOUCH (Smith & Nephew, Watford, United Kingdom) was used as a negative pressure wound therapy (NPWT) device (Fig. 1f, g). A cotton foam filler (Smith and Nephew, Watford, United Kingdom) was placed over the wound to prevent subcutaneous hematoma formation. Subsequently, a waterproof, airtight film was applied to cover the area (Fig. 1g). A round hole several centimeters in diameter was made in the center of this film, directly above the foam, to attach a soft port linked to the RENASYS TOUCH through a Y-connector, and the setup depended on the number of connected Salem Sump tubes (Fig. 1h). The cotton foam filler was replaced once weekly, and the wound was examined.

##### Postoperative antibiotics

**Fig. 2:**
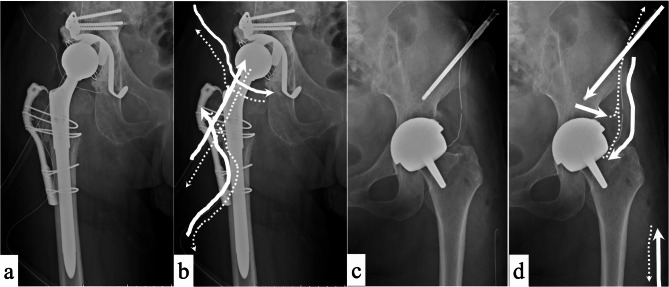
Continuous local antibiotic perfusion treatments. **(a).** Treatment for periprosthetic joint infection after revision total hip arthroplasty. It involves debridement with implant retention. One intra-soft tissue antibiotic perfusion device is placed distally and one proximally around the femur and acetabulum. An intra-joint antibiotic perfusion device is also placed in the hip joint, all perfused with local antimicrobials. (**b).** Antimicrobial pathways. White arrows indicate the routes of antimicrobial administration; white dotted lines indicate the collection of drainage fluid after perfusion. (**c).** Treatment following hip-resurfacing arthroplasty. The treatment is performed for periprosthetic joint infection in combination with debridement and implant retention. One intra-soft tissue antibiotic perfusion device is placed distally around the femur and one intra-joint antibiotic perfusion device is placed in the hip joint. In addition, one intramedullary antibiotic perfusion device is placed from the ala of the ilium, all perfused with local antimicrobials. (**d).** Administration and drainage details. White arrows indicate the route of antimicrobial administration, and white dotted lines indicate the collection of drainage fluid after antimicrobial perfusion. Drilling through the acetabulum allows for antimicrobial perfusion.

Gentamicin sulfate eradicates biofilms effectively in susceptible general bacteria and in methicillin-resistant bacteria^2,17^. Thus, for bacterial infections, regardless of the causative bacteria or resistance profile, dilutions of gentamicin sulfate, amikacine sulfate or arbekacin sulfate were prepared in saline at 1.2 or 2.0 mg/mL, respectively (Fig. 2a–d). These solutions were continuously administered at a rate of 2.0 mL/h/route for 24 h daily using a continuous precision pump through a Salem Sump tube. For fungal infections, micafungin sodium, which is effective for biofilms, was diluted in saline to 50 µg/mL and administered at the same rate and duration^18^. Notably, each route was flushed daily with 3.0–10.0 mL of gentamicin-diluted solution or saline solution to prevent clogging. The gentamicin diluent was stopped one day before the scheduled removal of the CLAP device, allowing the antibacterial agent or fluid to be collected in the tissue over a day. If CLAP administration was obstructed by tube clogging, the CLAP was removed. Tubes, pins, or devices could be easily removed from a patient’s bedside.

Postoperatively, a broad-spectrum systemic antimicrobial was used intravenously at first, and de-escalation was performed based on bacterial culture results. Anti-methicillin-resistant *Staphylococci* (MRS) antimicrobials were used in combination for the patients with the molecular diagnosis positive for MRS. Systemic antimicrobials were discontinued at the surgeon’s discretion once the serum inflammation improved. If the blood tests indicated persistent inflammation in chronic suppression owing to the patient’s condition, antibacterial medication was continued. All the complications due to CLAP use that required treatment adjustment or discontinuaction were documented.

#### Variables, outcome measures, data sources, and bias

The following characteristics were investigated: age at the time of surgery, sex, body mass index (kg/m^2^), follow-up duration since surgery, implant type, the host’s compromised medical history, causative organisms identified in cultures, and sensitivity to gentamicin sulfate. Patients who died over 1 year post-surgery were considered to have completed the follow-up period. Treatment details assessed included operative time, intraoperative blood loss, CLAP type (iSAP, iJAP, or iMAP), treatment duration until CLAP device removal, number of DAIR surgeries involving CLAP, types of antibacterial or antifungal agents used, hospitalization duration, length of postoperative systemic antibacterial treatment (duration of intravenous and oral administration), and activities of daily living 1 year postoperatively.

The primary endpoint was the implant survival rate post-surgery, with failure indicated by either the need for implant removal or replacement due to recurrent infection. The secondary endpoint was the presence or absence of adverse events including gentamicin or arbekacin levels in the blood at 3 days and 1 week postoperatively and immediately before the removal of the CLAP device. Adverse events assessed were renal impairment (increased blood creatinine level; renal function deterioration was defined as an increase in blood creatinine concentration of ≥ 0.30 mg/dL^19^), hepatic impairment (increased aspartate transaminase or alanine aminotransferase levels), pancytopenia, hearing impairment, visual impairment, skin impairment, and allergic symptoms. Serum assays for white blood cell counts and C-reactive protein levels were conducted immediately before surgery, and at 3 days, 1 week (± 1 day), 2 weeks (± 1 day), 3 weeks (± 1 day), 1 month (± 2 days), and 3 months (± 2 weeks) postoperatively.

#### Statistical analysis and Study size

All the PJI cases with or without CLAP were retrospectively analyzed. The collected items were compared between the CLAP group and the non-CLAP group, which was used as the historical control group. All the statistical analyses were performed using JMP Pro version 17.0 (SAS Institute, Inc., Cary, NC, USA). Statistical significance was determined using Student’s *t*-tests. Non-parametric Mann–Whitney tests were applied to data sets that were not normally distributed or were not of equal variance. Fisher’s exact test was used to analyze associations between categorical variables. Statistical significance was set at *p* < 0.05. Graphs were created in Prism 9 (MDF Co., Ltd., Tokyo, Japan), showing the interquartile range and the mean as a broken line.

#### Demographics and Description of the study population

The screening dataset included 24 patients (15 women and nine men) in the CLAP group and 10 patients (six women and four men) in the non-CLAP group. Of these patients in the CLAP group, two (a male and a female) died within a year post-surgery owing to factors unrelated to CLAP. We analyzed the data from the remaining 22 patients (14 women and eight men); two of them (nos. 8 and 17) who died of causes unrelated to the surgery over 1 year postoperatively without infection recurrence and were included in the study. In contrast, no cases in the non-CLAP group were lost to follow-up, and all 10 cases were included in the analysis.

## Results

### Complications in patients with PJI

All the participants met the major diagnostic criteria for PJI specified at the 2018 International Consensus Meeting^16^. The fistula was associated with prostheses in 10 patients in the CLAP group and in seven patients in the non-CLAP group, and the same bacteria or fungi were detected at two or more sites in 20 patients and in seven patients, respectively (Table 1). All patients in both groups met major criteria and were diagnosable with PJI. Notably, some patients did not undergo preoperative blood assays for D-dimer and erythrocyte sedimentation rates and lacked intraoperative tissue sample-based pathological diagnoses. No significant differences in the diagnostic criteria such as presence of fistula, CRP, D-dimer, and erythrocyte sedimentation rates were found between the groups.

**Table 1:** Characteristics and comparison of periprosthetic joint infection based on the diagnostic criteria of the 2018 international consensus meeting in the CLAP and the non-CLAP groups.

		CLAP (*n* = 22)	non-CLAP (*n* = 10)	*P* value (CI: 0.95)
Fistula (n)		10 (45.5%)	7 (70.0%)	0.446
Culture* (n)	≥ 2 1 0	20 (90.9%) 2 (9.1%) 0 (0%)	7 (70.0%) 2 (20.0%) 1 (10.0%)	0.2345
Phase (n)	Acute Chronic	11 (50.0%) 11 (50.0%)	8 (80.0%) 2 (20.0%)	0.109
CRP (mg/L)		102 ± 156 (range 1–291)	38 ± 46 (range 1–149)	0.416
D-dimer (µg/L)		4,836 ± 3,169 (range 1,400–12,500) (*n* = 14)	5,898 ± 3,870 (range 600–12,800) (*n* = 9)	0.614
ESR (mm/h)		56.6 ± 37.8 (range 5–130) (*n* = 12)	52.1 ± 30.7 (range 20–113) (*n* = 7)	0.833
Histology (n)	+ - NA	12 (54.5%) 2 (9.1%) 8 (36.4%)	6 (60.0%) 3 (30.0%) 1 (10.0%)	0.343
Purulence (n)	+ -	20 (90.9%) 2 (9.1%)	9 (90.0%) 1 (10.0%)	1.000

Periprosthetic joint infection scores are calculated based on the diagnostic criteria of the 2018 International Consensus Meeting. Preoperatively, variables including the fistula, culture, phase of infection (acute ≤ 4 weeks or chronic > 4 weeks from onset), serum C-reactive protein and D-dimer levels, and the erythrocyte sedimentation rate are evaluated; the histology and purulence are evaluated intraoperatively or postoperatively. Patients are diagnosed with periprosthetic joint infections based on the major diagnostic criteria of the presence of a fistula or a case in which the same bacteria are detected at two or more sites in the bacterial culture assay. If the major criteria are unmet, patients with a score ≥ 6 for the minor diagnostic criteria are diagnosed with periprosthetic joint infection (mean ± 1 standard deviation).

CLAP, continuous local antibiotic perfusion; CRP, C-reactive protein; ESR, erythrocyte sedimentation rate; NA, not available.

* Number of locations where culture is detected.

Table 2 presents the patients’ characteristics and surgical data. Body mass index was significantly lower in the CLAP group than in the non-CLAP group (*p* = 0.0384). Of the 22 patients in CLAP group, 12 had a medical history of an immunocompromised state: seven had diabetes, three had rheumatoid arthritis, one had multiple myeloma, and one had systemic lupus erythematosus. Of the 10 patients in the non-CLAP group, one patient had rheumatoid arthritis, and one had hemodialysis. Blood cultures of all patients except one in each of the two groups showed pathogenic bacteria or fungi. Methicillin-resistant bacteria were detected in 11 patients in the CLAP group and 5 patients in the non-CLAP group, *Candida* species in two only in the CLAP group. Sensitivity to gentamicin sulfate was observed in 11 patients, and one patient underwent two surgeries involving DAIR and CLAP in the CLAP group.

**Table 2:** Patient characteristics and surgical data.

		CLAP (*n* = 22)	non-CLAP (*n* = 10)	*P* value (CI: 0.95)
Age (years)		71.1 ± 13.8 (range 32–93)	64.1 ± 16.7 (range 22–82)	0.214
Sex (female, n)		14 (63.6%)	6 (60.0%)	0.844
Body Mass Index (kg/m^2^)		21.9 ± 4.1 (range 16.4–30.4)	27.4 ± 6.5 (range 19.8–37.8)	0.038*
Follow-up (months)		42.6 ± 31.5 (range 12–161)	56.8 ± 28.8 (range 28–114)	0.077
Implants (n)	BHA THA Revision HRA	8 (36.4%) 8 (36.4%) 5 (22.7%) 1 (4.5%)	1 (10.0%) 5 (50.0%) 4 (40.0%) 0 (0%)	
Medical history (n)	Diabetes Rheumatoid arthritis Others	7 (31.8%) 3 (27.3%) 2 (18.1%)	0 (0%) 1 (10.0%) 1 (10.0%)	0.069 1.000 0.333
Microorganism (n)	MSSA MRS *Streptococcus* *Corynebacterium* Others Not detected Mixed infection with *Candida*	4 (18.2%) 11 (50.0%) 2 (9.1%) 2 (9.1%) 2 (9.1%) 1 (4.5%) 2 (9.1%)	1 (10.0%) 5 (50.0%) 0 (0%) 1 (10.0%) 2 (20.0%) 1 (10.0%) 0 (0%)	
Sensitivity to gentamicin sulfate (n)	Susceptible Resistant NA	11 (50.0%) 10 (90.9%) 1 (4.5%)	6 (60.0%) 3 (30.0%) 1 (10.0%)	0.691

Items that cannot be retrospectively investigated from the medical records are recorded as not available (mean ± 1 standard deviation). The medical histories of only immunocompromised hosts are described.

CLAP, continuous local antibiotic perfusion; BHA, bipolar hip arthroplasty; THA, total hip arthroplasty; Revision, revision total hip arthroplasty; HRA, hip-resurfacing arthroplasty; MSSA, methicillin-sensitive *Staphylococcus aureus*; MRS, methicillin-resistant *Staphylococcus*; NA, not available; *, P value < 0.05.

Of the 23 surgeries, 18 utilized iSAP, 23 employed iJAP, and five used iMAP (Table 3). Antifungal agents were administered locally in two patients (nos. 5 and 12) due to fungal detection in cultures. We could not confirm from the medical records when oral systemic antimicrobial therapy was discontinued in three patients (nos. 1, 2, and 3). In another three patients (nos. 5, 12, and 16), antimicrobial medication was continued long-term, placing them in a state of chronic suppression.

**Table 3:** Operative characteristics of the participants.

S. No.	Operation time (min)	Blood loss (mL)	iSAP	iJAP	iMAP	CLAP duration (Days)	DAIR times	Perfused antibacterial or antifungal agents	Hospitalization (Days)	Duration of oral antibacterial or antifungal treatment (Weeks)	ADL
1	84	N/A		1		18	1	Gentamicin sulfate	88	Unknown*	T-cane
2	77	N/A	1	1		15	1	Gentamicin sulfate	48	Unknown*	Wheel-chair
3	NA	N/A	1	1	1	14	1	Arbekacin sulfate	43	16	Free-hand
4	117	N/A		1	1	16	1	Gentamicin sulfate	85	4	Free-hand
5	127	90	2	1		19	1	Gentamicin sulfate, micafungin sodium	47	Continued**	T-cane
6	142	N/A	1	1	1	16	1	Gentamicin sulfate	37	56	T-cane
7	150	110		1		8	1	Gentamicin sulfate	49	267	T-cane
8	60	N/A		1		20	1	Gentamicin sulfate, arbekacin sulfate	55	Unknown*	Circular walker
9	182	190		1		13	1	Gentamicin sulfate	48	90	T-cane
10	154	160	1	1		21	1	Gentamicin sulfate	75	24	Free-hand
11	85	10	1	1		9	1	Gentamicin sulfate	16	80	T-cane
12							2		120	Continued**	T-cane
First	211	1,550	1	1		15		Gentamicin sulfate			
Second	54	463	1	1		21		Gentamicin sulfate, micafungin sodium			
13	109	140	1	1	1	14	1	Gentamicin sulfate	20	93	Free-hand
14	137	280	1	1		14	1	Gentamicin sulfate	53	53	Wheel-chair
15	123	690	1	1		17	1	Gentamicin sulfate	58	352	Free-hand
16	109	780	1	1		15	1	Gentamicin sulfate	24	Continued**	T-cane
17	108	210	1	1		17	1	Gentamicin sulfate	26	177	T-cane
18	145	260	1	1		14	1	Gentamicin sulfate	24	48	T-cane
19	175	354	1	1		13	1	Gentamicin sulfate	39	144	Free-hand
20	294	640	1	1	1	14	1	Gentamicin sulfate	25	87	Free-hand
21	144	580	1	1		26	1	Gentamicin sulfate	63	364	Free-hand
22	122	222	1	1		8	1	Gentamicin sulfate	21	82	Free-hand
Mean	132 ± 53 (*n* = 22)	396 ± 375 (*n* = 17)				15.5 ± 4.2 (*n* = 23)			51.1 ± 26.2 (*n* = 23)		

Continuous local antibiotic perfusion and antibacterial treatments are administered in all cases (mean ± 1 standard deviation).

Items that cannot be retrospectively investigated from the medical records are recorded as not available.

CLAP, continuous local antibiotic perfusion; ADL, activities of daily living 1 year after surgery; DAIR, debridement, antibiotics, and implant retention; iJAP, intra-joint antibiotic perfusion; iMAP, intramedullary antibiotic perfusion; iSAP, intra-soft tissue antibiotic perfusion; N/A, not available.

* The antibacterial medication course is completed, but it is difficult to decipher the date from the medical records.

**Antibacterial medication continued and led to a state of chronic suppression, characterized by a quiescent infection without exacerbation.

### Implant survival after CLAP

The implant survival rate after DAIR surgery supplemented with CLAP was 90.9% (20 of 22 cases; Table 4), and without CLAP was 70.0% (7 of 10 cases). In two cases (nos. 9 and 16) in the CLAP group, the implants were removed and replaced because of recurrent peri-implant infection. Specifically, one patient underwent two-stage revision total hip arthroplasty (THA) for both the cup and stem 5 months later. The other underwent one-stage revision THA for the stem alone 4 months later. During these revision surgeries, CLAP was reintroduced, and local antimicrobial therapy was administered alongside systemic antimicrobial therapy, successfully resolving the infections in both cases. In three cases in the non-CLAP group, the implants were removed because of recurrent peri-implant infection. Two cases underwent revision total hip arthroplasty, while one patient could not receive a replacement.

**Table 4:** Implant survival rate in the continuous local antibiotic perfusion (CLAP) group and non-CLAP group, and post-CLAP treatment detailed outcome of implant preservation or removal in the CLAP group.

		CLAP (22 hips, 23 surgeries)	non-CLAP (10 hips, 10 surgeries)	*P* value (CI: 0.95)
Implant survival rate (%)		20 hips (90.9%)	7 hips (70.0%)	0.293
Number of surgeries maximal blood concentration of local antimicrobials	< 0.3 µg/mL 0.3–0.9 1.0–1.9 2.0–2.9 3.0 ≦ No measurement	5 (21.7%) 3 (13.0%) 6 (26.1%) 2 (8.7%) 1 (4.3%) 6 (26.1%)	– – – – – 10 (100%)	
Creatinine (mg/dL)	Before* After**	0.81 ± 0.23 0.79 ± 0.24	0.91 ± 0.67 0.88 ± 0.61	0.618 0.786
AST (IU/L)	Before* After**	22.0 ± 6.8 23.0 ± 18.9	23.9 ± 10.7 22.7 ± 8.9	0.376 0.424
ALT (IU/L)	Before* After**	14.7 ± 6.5 16.8 ± 20.8	22.1 ± 12.1 19.9 ± 11.5	0.081 0.084

ALT, alanine aminotransferase; AST, aspartate transaminase; CLAP, continuous local antibiotic perfusion.

None of the patients have pancytopenia, allergic symptoms, or hearing, visual, or skin impairments. Serum creatinine, aspartate transaminase, and alanine aminotransferase levels before and immediately after removal of the continuous local antibiotic perfusion system and investigation of the occurrence of drug-specific complications (mean ± 1 standard deviation) are shown. Items that cannot not be retrospectively investigated from the medical records are recorded as not available.

* Serum assays performed immediately before surgery.

**Serum assays performed immediately after removal of the continuous local antibiotic perfusion device.

**Fig. 3:**
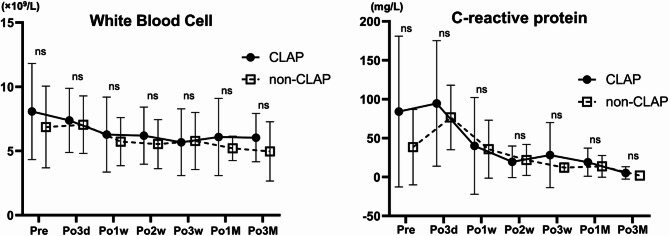
Serum assay results over time. Graphs showing the trends and comparison between the continuous local antibiotic perfusion (CLAP) group (23 surgeries, 22 patients) and non-CLAP group (10 surgeries, 10 patients) in white blood cell count and C-reactive protein levels from preoperative and postoperative serum assays across different time points. Values are measured immediately before the surgery and 3 days, 1 week, 2 weeks, 3 weeks, 1 month, and 3 months after surgery. Pre, before surgery; Po3d, 3 days after surgery; Po1w, 1 week after surgery; Po2w, 2 weeks after surgery; Po3w, 3 weeks after surgery; Po1M, 1 month after surgery; Po3M, 3 months after surgery.

Gentamicin sulfate was used as a high-concentration antimicrobial for CLAP due to its broad antibacterial spectrum, covering both Gram-positive and Gram-negative bacteria, and its concentration-dependent bactericidal effect. Gentamicin levels were not measured during the six DAIR surgeries with CLAP (nos. 1, 8, 9, 11, 12-first, and 12-second). Based on the KDIGO criteria, two cases (nos. 3 and 10) met the definition of Stage 1 acute kidney injury, with postoperative increases in serum creatinine of more than 0.3 mg/dL^19^. No cases met the criteria for Stage 2 or 3. However, serum creatinine levels promptly improved within 2 weeks in both cases. In one patient (no. 15), liver enzyme levels were elevated at the time of CLAP device removal. This patient, who had severe inflammation due to preoperative PJI and developed multiorgan failure due to postoperative acute respiratory distress syndrome, experienced rapid improvement in liver function after treatment in the intensive care unit. No other patient experienced liver or kidney dysfunction, and no complications specific to gentamicin or arbekacin sulfate were observed. The mean postoperative white blood cell counts and C-reactive protein levels decreased gradually without significant difference between the two groups (Fig. 3). Notably, although there was no significant difference, C-reactive protein levels decreased from the first to the second postoperative week, mildly increased at 3 weeks postoperatively only in the CLAP group, and continued to decrease until 3 months postoperatively.

## Discussion

The CLAP group exhibited a higher implant survival rate (90.9%) than both the control group (70.0%) and previous reports (58–83%)^4,20–22^, although the difference did not reach statistical significance. This improvement was attributed to the higher concentrations of gentamicin sulfate or arbekacin sulfate delivered by CLAP, which surpassed the MBEC and effectively destroyed biofilms and methicillin-resistant bacteria. Notably, some studies previously reported the efficacy of local antimicrobial infusion therapy^23–26^. Whiteside reported that local injection of high concentrations of antimicrobials into the artificial hip and knee joints through two Hickman catheters for 6 weeks resulted in high rates of infection control without adverse events^25,26^. This administration period is longer than the mean duration of CLAP therapy (15.5 days), and the lack of negative pressure drainage capability results in fluid retention and limited antimicrobial distribution during drug delivery. CLAP ensures effective antimicrobial perfusion delivery directly to the target joints, muscles, bones, and peri-implant areas, supported by the NPWT system and Salem Sump tubes with double lumen structure, unlike conventional methods. Successful outcomes depend on careful preoperative planning, appropriate CLAP device placement, and coordinated systemic antimicrobial administration to neutralize PJI in a single DAIR surgery. Accurate preoperative identification of the spread of infection is crucial and requires imaging modalities such as ultrasonography, computed tomography, magnetic resonance imaging, and bone scintigraphy to determine the optimal CLAP device placement^27^. In cases of infection recurrence, factors such as changes in bacterial species, insufficient antimicrobial perfusion for biofilm destruction, and residual infectious flora must be considered. For instance, in patient 12, CLAP was repeated to effectively quell the infection because of a shift in the causative organism from methicillin-resistant *Staphylococcus epidermidis* to *Candida albicans*.

The higher implant survival rate in DAIR surgeries with CLAP treatment for PJI is likely associated with its effective drug delivery system and optimal management of postoperative dead space. Negative pressure aspiration using an NPWT device for a few weeks postoperatively reduces dead space and bacterial colonization in coagulated blood, which is a common postoperative issue, especially in cases of notable bone and soft tissue loss. Unlike conventional drainage devices, NPWT maintains a constant negative pressure without interfering with the administration route, thereby preventing coagulation and bacterial growth. In addition, CLAP provides adjustable flow rates and versatility for use in unequipped facilities. However, one limitation is the need to remove the device if the clot obstructs the administration route.

Two patients with failed implant preservation in the CLAP group (nos. 9 and 16) had diabetes mellitus and infections caused by antibiotic-resistant bacteria (methicillin-resistant *S. aureus* and multidrug-resistant *P. aeruginosa*, respectively). In patient 9, inadequate local perfusion by a single iJAP device led to a two-stage revision THA with CLAP, which subsequently controlled the infection. In patient 16, a shift from *P. aeruginosa* to *Streptococcus* necessitated one-stage arthroplasty of the femoral component with CLAP. This instance suggests that bacterial shifts can compromise implant preservation, often leading to changes in systemic antimicrobials.

Arbekacin sulfate was used for treating PJI in methicillin-resistant cases (nos. 3 and 8) during early CLAP treatment phases, but gentamicin sulfate was preferred for most patients due to its effectiveness in biofilm destruction at high concentrations^5,17,28^. However, local use of gentamicin can lead to acute kidney injury^29,30^. Increased serum creatinine level was observed in two patients (nos. 3 and 10), but these levels improved after CLAP device removal; no severe kidney dysfunction occurred. Furthermore, no instances of non-dose-dependent adverse effects, such as eighth cranial nerve dysfunction (a known aminoglycoside side effect), were noted. Blood gentamicin levels were maintained below 1.0 µg/mL^31,32^, although some patients (nos. 2–4, 6, 10, 13, 14, 16, and 17) exceeded this threshold, highlighting the need to monitor and adjust blood antimicrobial levels during CLAP treatment to prevent acute kidney injury. Adjustments, such as reducing the flow rate and dose concentration or discontinuing the infusion immediately, can be made easily if high antimicrobial concentrations are detected or complications arise.

Postoperative serum C-reactive protein levels increased slightly at 2–3 weeks, whereas white blood cell counts continued to decrease after CLAP treatment. The increase in C-reactive protein levels after CLAP device removal was likely attributable to the reabsorption of exudate accumulated around the implant. Fungal PJI, with a DAIR success rate of only 18%^33^, presents notable treatment hurdles, particularly when two-stage revision is impractical because of extensive bone loss or megaprosthesis^34^. In this study, two patients with fungal PJI (nos. 5 and 12) required implant preservation. A concentrated local infusion of micafungin sodium, known for its antibiofilm properties, was administered through the CLAP^35–37^. Continuous micafungin administration successfully suppressed infection and preserved the implants. Furthermore, combining CLAP with antifungal agents in DAIR procedures may prevent fungal PJI. Potential complications, including hemolysis-associated thrombosis, kidney injury, and neutropenia, were not observed. Larger studies are warranted to confirm the safety of sodium micafungin in treating PJI. After establishing its efficacy and safety, CLAP could become a pioneering drug delivery system for managing bacterial and fungal PJI.

This study had some limitations, including a lack of some data and a short follow-up period for some cases. While patients continued follow-up visits at our hospital after being transferred to rehabilitation facilities, information from the latter could not be fully obtained. We plan to follow up on more cases of PJI and conduct comparative studies with conventional methods. Further research is required to ascertain whether the concentration and flow rate of antimicrobial agents should be adjusted for different cases.

## Conclusions

Adding CLAP to DAIR surgery shows promising results for treating PJI. However, as two patients experienced elevated serum creatinine levels, monitoring the blood concentrations of gentamicin is recommended in order to prevent severe adverse events. Further evaluation of outcomes in patients with PJI treated with CLAP may provide a valuable alternative treatment option.

## Data Availability

Most of the data are presented in this paper. The other data that support the findings of this study are available from the corresponding author upon reasonable request.
